# A Virtual Hospital Model of Care for Low Back Pain, Back@Home: Protocol for a Hybrid Effectiveness-Implementation Type-I Study

**DOI:** 10.2196/50146

**Published:** 2024-02-22

**Authors:** Alla Melman, Min Jiat Teng, Danielle M Coombs, Qiang Li, Laurent Billot, Thomas Lung, Eileen Rogan, Mona Marabani, Owen Hutchings, Chris G Maher, Gustavo C Machado, Back@Home Investigators

**Affiliations:** 1 Sydney Musculoskeletal Health The University of Sydney and Sydney Local Health District Camperdown Australia; 2 RPA Virtual Hospital Sydney Local Health District Sydney Australia; 3 The George Institute for Global Health University of New South Wales Sydney Australia; 4 School of Public Health Faculty of Medicine and Health The University of Sydney Sydney Australia; 5 Department of Medicine Canterbury Hospital Sydney Local Health District Sydney Australia; 6 See Acknowledgments

**Keywords:** length of stay, back pain, musculoskeletal pain, telemedicine, hospital-based home care, mobile phone, home care, virtual care, remote care, virtual hospital, pain, telehealth, eHealth, musculoskeletal, implementation, model of care, back, cost, economic, readmission, hospital stay

## Abstract

**Background:**

Low back pain (LBP) was the fifth most common reason for an emergency department (ED) visit in 2020-2021 in Australia, with >145,000 presentations. A total of one-third of these patients were subsequently admitted to the hospital. The admitted patient care accounts for half of the total health care expenditure on LBP in Australia.

**Objective:**

The primary aim of the Back@Home study is to assess the effectiveness of a virtual hospital model of care to reduce the length of admission in people presenting to ED with musculoskeletal LBP. A secondary aim is to evaluate the acceptability and feasibility of the virtual hospital and our implementation strategy. We will also investigate rates of traditional hospital admission from the ED, representations and readmissions to the traditional hospital, demonstrate noninferiority of patient-reported outcomes, and assess cost-effectiveness of the new model.

**Methods:**

This is a hybrid effectiveness-implementation type-I study. To evaluate effectiveness, we plan to conduct an interrupted time-series study at 3 metropolitan hospitals in Sydney, New South Wales, Australia. Eligible patients will include those aged 16 years or older with a primary diagnosis of musculoskeletal LBP presenting to the ED. The implementation strategy includes clinician education using multimedia resources, staff champions, and an “audit and feedback” process. The implementation of “Back@Home” will be evaluated over 12 months and compared to a 48-month preimplementation period using monthly time-series trends in the average length of hospital stay as the primary outcome. We will construct a plot of the observed and expected lines of trend based on the preimplementation period. Linear segmented regression will identify changes in the level and slope of fitted lines, indicating immediate effects of the intervention, as well as effects over time. The data will be fully anonymized, with informed consent collected for patient-reported outcomes.

**Results:**

As of December 6, 2023, a total of 108 patients have been cared for through Back@Home. A total of 6 patients have completed semistructured interviews regarding their experience of virtual hospital care for nonserious back pain. All outcomes will be evaluated at 6 months (August 2023) and 12 months post implementation (February 2024).

**Conclusions:**

This study will serve to inform ongoing care delivery and implementation strategies of a novel model of care. If found to be effective, it may be adopted by other health districts, adapting the model to their unique local contexts.

**International Registered Report Identifier (IRRID):**

PRR1-10.2196/50146

## Introduction

### Background

Low back pain (LBP) was the fifth most common reason for an emergency department (ED) visit in 2020-2021 in Australia, with more than 145,000 presentations [1]. One-third of these patients were subsequently admitted to hospital. The current annual admission rates for spinal conditions are high in Australia, at 465 per 100,000 population, compared to 219 in the United Kingdom, 197 in the Netherlands, and 142 in Canada [2]. Admission rates following LBP presentations to ED are higher in Australia [3,4] than have been reported in the United States [5]. The potential contributors to high admission rates are differences in case mix [6], patient expectations of hospital admission when experiencing high levels of distress [7], and lack of alternative pathways for prompt pain management. These hospitalizations pose a significant burden on the health care system. In Australia, for example, admitted patient care of LBP lasts an average of 9 days and costs Aus $15,000 (US $10,000) per admission [8].

Most patients admitted to the hospital with a primary LBP diagnosis do not have a serious underlying condition and there is evidence that admissions carry the risk of harm. Our recent medical record review of 1982 admissions found that 57% of inpatients with provisionally diagnosed musculoskeletal LBP in ED were discharged with this same diagnosis [9]. Bed rest, as typically occurs with traditional hospital admission, is not recommended in LBP guidelines [10] as it can delay recovery. A recent study showed that 23% of LBP admissions had opioid-related complications and other serious events such as falls in hospital (4%) and hospital-acquired infections (1.4%) [11]. These patients could be diverted to more cost-effective and safer alternate clinical pathways.

Virtual hospitals have been proposed as a potential clinical pathway for people with LBP [12], to facilitate early discharge. Interviews with people admitted for acute LBP have identified that returning home as soon as possible is a key patient priority; however, patients fear a lack of support if discharged home [13]. There is also evidence from other conditions that virtual hospitals are cost-effective. Systematic reviews of “early supported discharge” have been shown to safely reduce the length of the hospital stay in adults with a range of medical conditions [14]. A recent US trial of virtual hospital admission for mixed acute medical conditions showed 38% lower costs for virtual hospital patients compared to traditional admissions, and reduced use of laboratory tests, imaging, and consultations [15]. The virtual hospital cohort had higher levels of patient satisfaction and lower rates of adverse events [15].

We currently lack alternatives to traditional hospital admission for patients with musculoskeletal LBP who present to Australian EDs and require acute clinical care. A virtual hospital has been implemented in Sydney Local Health District, Australia for COVID-19–positive patients and other patient cohorts [16], caring for over 16,000 patients to date [17]. In the virtual hospital, clinicians use technologies for remote patient monitoring and management [12]. Monitoring via daily clinician contact is designed to reduce representation to the ED while providing clinical support and pain management until the patient is able to link in with and attend outpatient services. Given the higher rates of potentially serious pathology in this cohort (compared to primary care), it is also a form of safety netting, allowing prompt escalation if required. Physical activity monitoring is designed to substitute regular reminders to mobilize as would be delivered on a traditional ward and encourage patients to slowly upgrade physical activity. A virtual hospital service, however, is yet to be formally evaluated in patients with musculoskeletal LBP.

Alongside the diagnostic challenge of LBP, is the difficulty of discharging patients home when safe mobility is not yet achieved. Some patients may still require short-term traditional hospital admission to allow for effective analgesia to facilitate mobility. Hence, in this study, we will evaluate the effectiveness of an early-supported discharge virtual hospital model of care, with traditional hospital length of stay as the primary outcome.

### Aims

The main aims of the Back@Home study are to assess the effectiveness of a virtual hospital model of care for LBP on health service outcomes (eg, length of admission), patient-reported outcomes (eg, satisfaction with care), and costs. The secondary aims are to evaluate the acceptability and appropriateness of the virtual hospital model of care for LBP, as well as the feasibility and fidelity of our multifaceted implementation strategy.

## Methods

### Study Design

This is a hybrid effectiveness-implementation type-I study [18]. This study design will allow us to assess the effectiveness of a new virtual hospital model of care for LBP on health services outcomes while assessing the feasibility and acceptability of the new model and our multifaceted implementation strategy, as described by Curran et al [18]. We have used the guidance for conducting implementation trials by Pearson et al [19] and Proctor et al [20] to design this study.

### Setting

This study will be conducted at 3 public hospitals in Sydney, New South Wales, Australia. The hospitals have a combined 169,000 ED attendances per year, with 650,000 total “bed days” available annually, averaging 1530 inpatients per day [21].

### Population

To identify the study population, Systematized Nomenclature of Medicine Clinical Terms-Australian (SNOMED-CT-AU) diagnosis codes will be used to select patients in the ED aged 16 years and older with a primary discharge diagnosis related to musculoskeletal LBP. Those with an ED diagnosis code of a “serious” LBP condition (eg, vertebral fracture, spinal abscess, and cauda equina syndrome) will be excluded. We will then use inpatient discharge diagnosis codes (*International Classification of Diseases, Tenth Revision, Australian Modification* [*ICD-10 AM*]) to classify the LBP admissions as “serious” or “musculoskeletal” (ie, nonspecific LBP and lumbosacral radicular pain). Only data from admissions where inpatient discharge diagnosis codes are musculoskeletal LBP admissions will be evaluated.

Patients who presented to the ED with a primary diagnosis related to musculoskeletal LBP and were discharged without admission into a short stay or inpatient unit will be included in the data analysis to determine rates of hospital admission.

### Intervention

**Figure 1:**
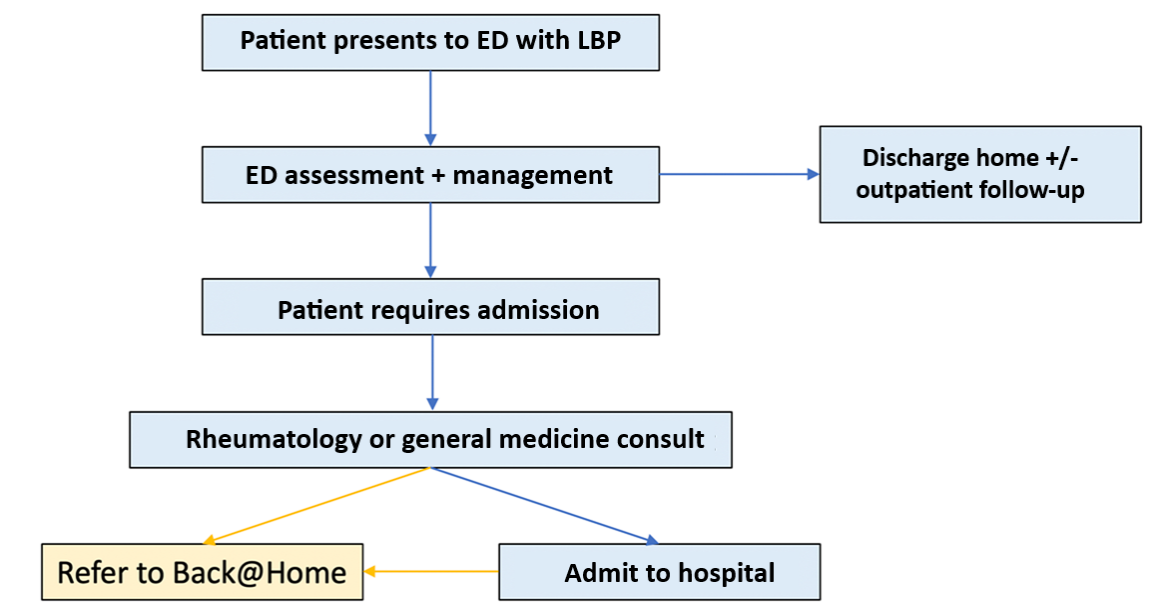
Virtual low back pain service workflows. ED: Emergency Department; LBP: low back pain.

Patients with LBP requiring admission in ED short stay or inpatient units will be assessed by senior ED staff. Eligible patients will be referred to the “Back@Home” virtual LBP service by the ED medical officer, in consultation with local rheumatology or general medicine admitting teams. The eligibility criteria for the virtual hospital are people aged 16 years and older diagnosed with musculoskeletal LBP, with or without radicular pain, requiring a higher level of clinical support above standard discharge home (see Figure 1).

Back@Home, a service run by Royal Prince Alfred Hospital (RPA) Virtual Hospital, will provide virtual “Hospital in the Home” care, including home visits, video calls, and remote monitoring. Eligible patients already admitted to the inpatient ward will also be referred to “Back@Home” to facilitate earlier discharge. Patients will be remotely monitored from home, and have 24/7 access to hospital-based clinicians through a “virtual” care center. An escalation pathway will be available if a patient’s condition deteriorates at home, and traditional admission, expedited imaging, or intervention is deemed necessary.

Medical care will be provided by virtual hospital physicians and nurses, with consultations from rheumatology specialists provided as required. All patients will be virtually assessed and treated by a physiotherapist, with additional assessments provided by occupational therapists, psychologists, and social workers as required.

Videoconferencing calls will be used to collect clinical observations and provide care. If required, patients will receive home visits from a physiotherapist [17]. The need for home visits will be decided by the multidisciplinary team (physician, nurse, and physiotherapist) during daily clinical reviews, in response to patient needs. If a patient appears to not be coping well at home and is at risk of ED representation, a home visit will be scheduled.

Remote monitoring of physical activity, such as step count, will be enabled via an activity tracker. The wearable device used (Garmin Vivovit 4) is a validated method of recording sleep cycles and sedentary and active time in community settings [22,23]. Patients will be able to report activity measures to their virtual physiotherapist during daily video calls, assisting in goal setting and behavioral health coaching.

Care will be provided through a variety of technologies as appropriate. SMS, telephone calls, videoconferencing calls (Zoom; Zoom Video Communications), patient information sheets emailed directly to patients, as well as access to the “Physitrack” smartphone app for the provision of health information content and exercise programs.

Traditionally admitted patients will receive usual clinical care, with a referral for physiotherapy mobility assessment if required, as per standard ward protocols. They will be eligible for referral to Back@Home to facilitate early supported discharge, if appropriate.

### Implementation Strategy

**Figure 2:**
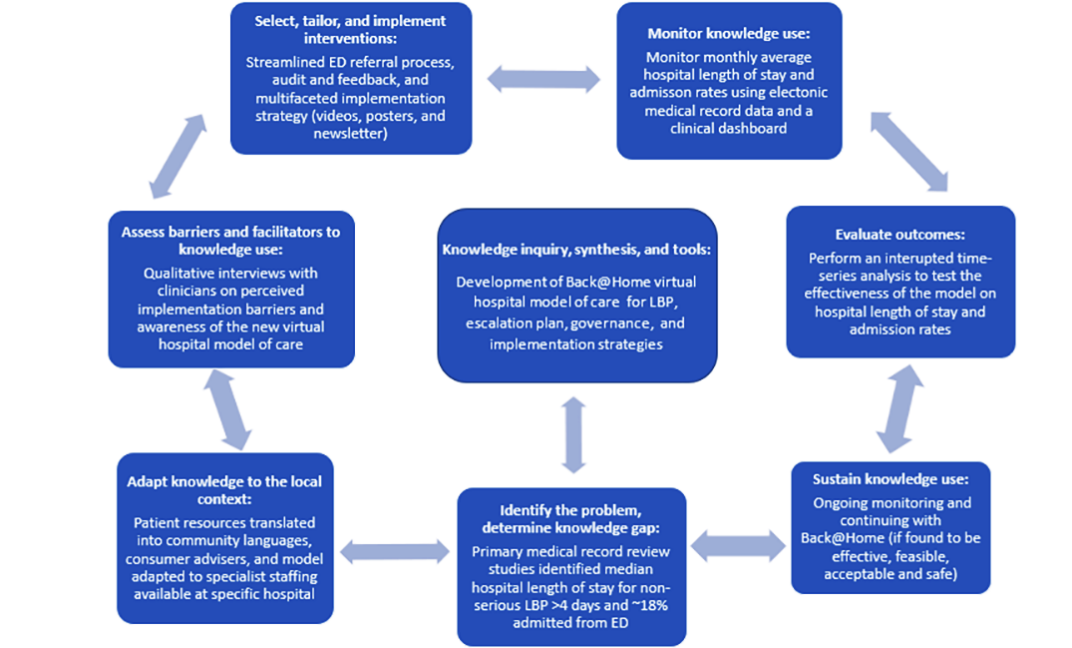
Knowledge to action framework. ED: Emergency Department; LBP: low back pain.

The implementation strategy will last for 3 months at each site. The “Knowledge to Action Framework” [24] (see Figure 2) was used to guide a research program to develop and evaluate the implementation of Back@Home. The framework structure includes identifying a clinical problem, and then developing evidence-based potential solutions while adapting these to the local context, evaluating the solution, and monitoring outcomes.

Semistructured interviews with hospital clinicians regarding perceived barriers and facilitators to implementation have informed the current design of the model of care [7]. Based on clinicians’ input, the Back@Home model of care will incorporate the loan of internet-enabled devices, health care interpreters, and written resources translated into community languages to facilitate more equitable access to care for marginalized groups. Feedback was provided by the Director of Aboriginal Health and the Aboriginal Cultural Support Team. Consultation on the model was also provided by ED medical officers, rheumatology, general medicine, and allied health departments, as well as nurse practitioners and social workers.

We will explore additional local barriers to implementation, design, and implement strategies to address these barriers, and monitor clinical practice and clinician behavior change. The evaluation will determine the effectiveness of the model of care, and if found effective, we will develop strategies to maintain health care system changes (see Figure 1).

To increase the uptake of the Back@Home model of care, we plan to use several implementation strategies that have been previously shown to be effective in changing professional behavior. First, staff training will be delivered by Back@Home investigators (MJT, ER, MM, and OH) at scheduled staff education sessions attended by physiotherapists, nurses, registrars, trainees, and consultants in ED and general medicine. Sessions will last 15 minutes and be delivered before implementation and at 1 and 3 months post implementation. Training will describe the process of referral to virtual care, deliver updates on implementation progress, and provide a forum to raise concerns or questions. Second, key local opinion leaders [25], in the form of “staff champions” will be provided with training. These staff will remind all clinical staff about Back@Home as an option for patients with musculoskeletal LBP requiring admission and will be identified by wearing a badge with the Back@Home logo. The ED primary care physiotherapist will play a key role in championing the Back@Home model of care. Third, a summary card containing admission criteria and contact details for the virtual hospital will be provided to relevant staff, which can be attached to staff lanyards for easy access (if permitted). This printed material will be used to reinforce the content of educational sessions [26,27]. Fourth, a monthly email update will be sent to ED, general medicine, and rheumatology staff regarding the length of hospital stay and rates of admission to traditional hospitals. Audit and feedback strategies have been shown to be effective in influencing health professionals’ behavior [28], and in supporting back pain model of care implementation strategies [29].

### Evaluation

#### Health Service Outcomes

The health service outcomes include (1) monthly mean length of hospital stay (ED short stay units and inpatient units) in those admitted as a traditional inpatient following an ED presentation for musculoskeletal LBP (primary outcome; Table 1); (2) monthly proportion of ED musculoskeletal LBP presentations that result in traditional hospital admissions (ED short stay or inpatient units); (3) monthly proportion of admitted patients representing to traditional hospital, including representations to the ED within 48 hours since discharge and readmissions to an inpatient unit within 28 days; and (4) mean and total hospital health care costs per month, for patients admitted to traditional and virtual hospitals.

**Table 1:** Evaluation plan.

Outcome dimension	Outcome	Outcome definition	Data source	Data collection and analysis period
Service	(1) Length of hospital stay; (2) inpatient admission; (3) rerepresentations; and (4) readmissions to inpatient units	(1) Monthly mean length of hospital stay (ED^a^ short stay units and inpatient units) in those admitted as traditional inpatients following an ED presentation for musculoskeletal LBP^b^; (2) monthly proportion of ED musculoskeletal LBP presentations that result in traditional hospital admissions (ED short stay or inpatient units); (3) monthly proportion of admitted patients representing to a traditional hospital, including representations to the ED within 48 hours since discharge; and (4) monthly proportion of admitted patients who are readmissions to an inpatient unit within 28 days.	1-4: eMR^c^	Baseline: 2016 January to 2023 February; T1^d^: 2023 August; T2^e^: 2024 February
Implementation	(1) Acceptability; (2) appropriateness; (3) feasibility; and (4) fidelity	(1-3) acceptability, appropriateness, and feasibility of the Back@Home “Model of Care” and implementation strategies (posters, staff training, videos, and newsletter); and (4) fidelity of the delivery of implementation strategies as planned.	(1-3) Semistructured interviews with clinicians and patients; (4) logbook of implementation delivery	T1: 2023 August
Patient	(1) Pain intensity; (2) physical function; (3) satisfaction with care; and (4) adverse events	(1) Numeric rating pain scale (0-10); (2) PROMIS^f^ Physical Function-6a; (3) global satisfaction with care for traditional and virtual admissions (0-10 points) and patient-reported experience measures as routinely collected by the virtual hospital (26-item survey); and (4) proportion of patients experiencing any AE^g^; frequency of AEs for virtual admissions	(1-3) Patient survey; (4) eMR	Collected at 2 and 4 weeks post ED presentation or during admission (adverse events), analyzed at T1 and T2
Process	(1) Diagnostic tests ordered; (2) pain medicines used; (3) video and phone calls; (4) home visits received; (5) Physitrack app use; and (6) Physitrack and activity tracker usefulness	(1) Proportion of virtually admitted patients receiving diagnostic tests; (2) proportion of virtually admitted patients prescribed specific medicines; (3) number and frequency of video consultations with virtual hospital clinical staff; (4) number and frequency of clinician home visits; (5) usage rates of the Physitrack app by patients (log-ins, viewing, and marked completion of exercise program); and (6) patient-reported experience measures questions 7 and 9 (5-point scale).	(1-4) eMR; (5) Physitrack app; and (6) Patient-Reported Experience Measure survey	Collected during virtual admission, analyzed at T1 and T2
Health economic	Hospital admission costs	Cost of delivering virtual care compared to inpatient care for nonserious low back pain.	eMR and finance reporting systems	Collected during admission and analyzed at T1 and T2

^a^ED: Emergency Department.

^b^LBP: low back pain.

^c^eMR: electronic medical record.

^d^T1: 6 months post implementation.

^e^T2: 12 months post implementation.

^f^PROMIS: Patient-Reported Outcomes Measurement Information System.

^g^AE: adverse event.

#### Implementation Outcomes

Implementation outcomes discussed in clinician and patient interviews will include (1) acceptability, appropriateness, and feasibility of the model of care to clinicians and the health service evaluated at 6 months using semistructured interviews [30] and (2) fidelity to planned implementation at 6 months using a logbook of implementation.

#### Patient-Reported Outcomes

Patient-reported outcomes will be collected at 2 and 4 weeks following hospital admission from patients admitted to traditional and virtual hospitals. Outcomes include average pain intensity in the past week (Numeric Rating Scale, range 0-10), Patient-Reported Outcomes Measurement Information System (PROMIS) Short Form—Physical Function 6b (range 6-30), and global rating of satisfaction with care (range 0-10).

#### Process Measures (Virtual Hospital Admissions Only)

First, the proportion of virtually admitted patients receiving diagnostic tests, by type: laboratory tests; lumbar imaging tests: plain radiography (x-ray), computerized tomography scan, magnetic resonance imaging; and per month.

Second, the proportion of virtually admitted patients prescribed specific medicines, per month. Pain medicines will be classified according to the Anatomical Therapeutic Chemical (ATC) classification systems. The drug dosage regimens will also be collected for the following groups of medicines: simple analgesics (ie, paracetamol); nonsteroidal anti-inflammatory drugs; weak opioids (eg, tramadol and codeine); strong opioids (eg, oxycodone and morphine); muscle relaxants; benzodiazepines; antiepileptics; antidepressants; and corticosteroids.

Third, the usage rates of the Physitrack app by patients (log-ins, viewing, and marked completion of exercise program). Fourth, the number and frequency of video consultations with virtual hospital clinical staff. Fifth, the number and frequency of face-to-face consultations (home visits) with staff. Sixth, the number and frequency of escalations of care (patient transferred to hospital), and adverse events. Seventh, the patient-reported experience measures as routinely collected by the virtual hospital, with additional questions relevant to Back@Home patients (26-item survey).

### Data Collection

#### Health Service Outcomes

We will extract primary and secondary health care use data from electronic medical records. Data extracted will include patient demographics (eg, age, gender, and postcode), ED presentation and inpatient admission or discharge date, length of ED and admission stay, discharge primary and secondary Systematized Nomenclature of Medicine (SNOMED)/*ICD-10* codes, diagnostic tests used (eg, laboratory tests and imaging), pain medicines received, specialist and allied health consultations, and health care costs. Hospital-acquired complications or adverse events will be identified via Classification of Hospital Acquired Diagnoses (CHADx) codes present in *ICD-10 AM* data [30]. Patient medical record number, encounter or visit identifier will be replaced by study ID as part of the data extraction process, resulting in deidentified data.

#### Implementation Outcomes

Semistructured interviews will be conducted with key clinical staff and hospital managers at the implementation site, as well as those involved in delivering virtual care. Purposeful sampling will aim for input from physiotherapists, nurses, ED medical officers, and rheumatologists. Patients who have experienced Back@Home virtual care will also be asked to participate (approved by Sydney Local Health District X21-0094 and 2021/ETH00591).

The fidelity of training-related implementation delivery will be assessed via a logbook of staff training, noting the number of sessions delivered, session delivery mode, and number of attendees.

#### Patient-Reported Outcomes

Patients with musculoskeletal LBP as the primary reason for admission (to traditional or virtual hospitals) will be eligible to complete the patient-reported outcome survey. Before implementation, we will use electronic medical records to identify a cohort that would have likely been eligible for virtual admission if it were available. This will include traditionally admitted patients with a Waterlow Mobility Score of 0-3 (not bed-bound) on admission, with a diagnosis of musculoskeletal LBP. All virtually admitted patients with LBP will be eligible. Automated text message invitations will be sent to eligible patients (via REDCap [Research Electronic Data Capture; Vanderbilt University] and Twilio) containing a link to an web-based survey, at 2 and 4 weeks following the ED presentation. One reminder message will be sent to nonresponders, and patients who do not respond to the text message will be followed up with a telephone call and offered the opportunity to ask any questions regarding participation or complete the survey by telephone. This process will be used to maximize the response rate of the surveys and has been proven to be feasible [31].

### Statistical Analysis Plan

#### Health Service Outcomes

Time-series trends during a retrospective 48-month period before the implementation of the new model of care will be compared with trends during a 12-month postimplementation period. Preliminary analysis will be conducted at 6 months post implementation. We will display the length of admissions as monthly averages and construct a plot of the observed and expected lines of the trend based on the preimplementation period. Linear segmented regression will identify changes in the level and slope of fitted lines. The standard interrupted time series model that will be used is



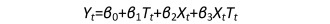



In this equation, *Y_t_* is the outcome variable (eg, length of admission) measured at each equally spaced time point *t* (monthly). *β*_0_ represents the intercept or starting level of the outcome variable and *β*_1_, the slope or trajectory of the outcome variable until the introduction of the intervention. *T_t_* represents the month of the initial ED presentation with *T*_0_ representing the first month (2017 January). *β*_2_ represents the change in the level of the outcome that occurs in the period immediately following the introduction of the intervention (compared with what would have happened in the absence of the intervention). *β*_3_ represents the difference between preintervention and postintervention slopes of the outcome. Thus, we look for significant differences in *β*_2_ to indicate an immediate treatment effect, and in *β*_3_ to indicate a treatment effect over time.

Data will be analyzed using SAS software (version 9.4; SAS Institute). Descriptive statistics will be used for patient demographics and clinical characteristics. Categorical variables will be described with frequencies (%) and continuous variables will be described with means and SDs.

To account for fluctuations in preimplementation hospital admissions due to COVID-19 pandemic–related restrictions, a time series analysis will account for these time periods. Restricted periods will be considered as February 1 to June 30, 2020; June 1 to December 1, 2021; and January 1 to May 1, 2022.

#### Patient-Reported Outcomes

Patient-reported secondary outcomes will include pain, physical function, and satisfaction with care at 2 and 4 weeks following admission. Group sample sizes of 100 eligible patients admitted to traditional hospital wards and 100 patients admitted to RPA Virtual Hospital, will be required to achieve 80% power to detect a noninferiority 1-point difference in patient-reported outcomes for satisfaction with care (0-10 continuous scale) between groups using a 1-sided, 2-sample equal-variance *t* test. The margin of noninferiority will be –1. The actual difference between the means will be assumed to be 0. The significance level (α) of the test will be set at .025. The data will be drawn from populations with an SD of 2.5 in both groups.

### Ethical Considerations

This investigation will be conducted in full compliance with the Declaration of Helsinki. Ethics approval has been granted by the Ethics Review Committee of Royal Prince Alfred Hospital (protocol X21-0278 and 2021/ETH10967). A waiver of consent has been approved for routinely collected data sourced from electronic medical records. Study data will be fully deidentified, to protect patient, clinician privacy, and confidentiality. Informed consent will be collected for all patient-reported outcomes. No compensation will be offered for participation.

### Patient and Public Involvement

The virtual hospital model of care was developed with the assistance of semistructured interviews with clinicians from several disciplines across 3 metropolitan hospitals. Clinicians from the departments of physiotherapy, rheumatology, and emergency medicine participated in a co-design process with researchers and administrators at RPA Virtual Hospital at Sydney Local Health District.

### Economic Evaluation Plan

An economic evaluation will be undertaken from the health system perspective. Costs associated with implementing Back@Home virtual care and patients’ health service use will be measured, using a combination of electronic medical records and financial trial records. Intervention-related costs include the development of training materials and salaried time of staff attending training workshops, changes in workload for staff delivering virtual care and costs related to information technology support and maintenance of the virtual network. Health service costs will be measured using Independent Health and Aged Care Pricing Authority national weighted activity units for admitted inpatient hospital and ED presentations; prescription and over-the-counter medications using the Pharmaceutical Benefits Scheme and pharmacy prices. All costs will be reported in Australian dollars. Where necessary, costs will be converted to 2023 prices using the health consumer price index published by the Australian Bureau of Statistics. Costs and effects will be discounted where appropriate.

We plan to conduct a cost-effectiveness analysis alongside this time series analysis with data from the intervention site. Similar to the time series model for health service outcomes, a generalized linear model with a gamma distribution and logarithmic link function will be used for the segmented regression analysis of health care costs. An incremental cost-effectiveness ratio will be calculated for the health services outcomes: length of stay and hospital admissions. This will be presented as incremental costs per bed day avoided and incremental cost per hospital admission avoided. Nonparametric bootstrapping with 5000 replications will be used to estimate the 95% CIs around the incremental cost and effect pairs for both health services outcomes. These will be presented on an incremental cost-effectiveness plane. We also plan to explore the cost-effectiveness using the patient outcomes from this study. If the patient outcomes analysis shows noninferiority as hypothesized, we will perform a cost-minimization analysis by comparing the cost between the control and intervention phases.

## Results

We will collect data from January 1, 2017, to September 30, 2024, and a 60-month period within this time frame will be used in the analysis, determined by the implementation schedule for the new model of care. Back@Home participant data will be collected from February 2023, following roll out of the service. For primary and secondary health service outcomes, we expect to collect data from approximately 12,500 patients with musculoskeletal LBP attending the 3 study EDs over a 5-year period, via the electronic medical record system. Interim process evaluation and implementation outcomes are expected to be published in early 2024, and the final study results are expected to be published in 2025. As of December 6, 2023, a total of 108 patients have been cared for through Back@Home. A total of 6 patients have completed semistructured interviews regarding their experience of virtual hospital care for nonserious back pain. All outcomes will be evaluated at 6 months (2023 August) and 12 months post implementation (2024 February).

## Discussion

This study will investigate the implementation of a novel model of care for nonserious back pain, delivered through a virtual hospital. Process evaluation will be used to inform further iterations of the service, guided by the Knowledge To Action framework [24]. We anticipate that the feasibility of implementing Back@Home will be demonstrated, along with the acceptability of the model of care to clinicians and patients and cost-effectiveness. Additionally, we hypothesize that patient-reported outcomes (pain and satisfaction with care) will be noninferior to traditional hospital admission. Interpretation of the patient-reported outcomes may be limited by response rate and strategies have been planned to optimize the response rate.

The interrupted time-series design has been commonly used to evaluate the impact of new patient pathways on admission rates, length of stay, and ED representation [32-34] for a variety of health conditions. We hypothesize that following the introduction of Back@Home, hospital length of stay, and admission rates for LBP will be reduced, compared to preimplementation measures. If proven to be safe, acceptable, effective, and cost-effective, virtual care for nonserious back pain could be expanded to other health districts in New South Wales, and potentially other states. Implementation in other jurisdictions would depend on staffing and technological resources to deliver virtual care.

## Abbreviations

ATC: Anatomical Therapeutic Chemical
CHADx: Classification of Hospital Acquired Diagnoses
ED: emergency department
ICD-10 AM: International Classification of Diseases, Tenth Revision, Australian Modification
LBP: low back pain
PROMIS: Patient-Reported Outcomes Measurement Information System
REDCap: Research Electronic Data Capture
RPA: Royal Prince Alfred Hospital
SNOMED: Systematized Nomenclature of Medicine
SNOMED-CT-AU: Systematized Nomenclature of Medicine Clinical Terms-Australian

## Data Availability

The data sets generated and analyzed during this study are available from the corresponding author on reasonable request.
