# Correction: A Virtual Hospital Model of Care for Low Back Pain, Back@Home: Protocol for a Hybrid Effectiveness-Implementation Type-I Study

**DOI:** 10.2196/60298

**Published:** 2024-05-16

**Authors:** Alla Melman, Min Jiat Teng, Danielle M Coombs, Qiang Li, Laurent Billot, Thomas Lung, Eileen Rogan, Mona Marabani, Owen Hutchings, Chris G Maher, Gustavo C Machado, Back@Home Investigators

**Affiliations:** 1 Sydney Musculoskeletal Health The University of Sydney and Sydney Local Health District Camperdown Australia; 2 RPA Virtual Hospital, Sydney Local Health District Sydney Australia; 3 The George Institute for Global Health, University of New South Wales Sydney Australia; 4 School of Public Health, Faculty of Medicine and Health, The University of Sydney Sydney Australia; 5 Department of Medicine, Canterbury Hospital, Sydney Local Health District Sydney Australia; 6 See Acknowledgments Camperdown Australia

In “A Virtual Hospital Model of Care for Low Back Pain, Back@Home: Protocol for a Hybrid Effectiveness-Implementation Type-I Study” (JMIR Res Protoc 2024;13:e50146) the authors noted the following errors.

In the originally published article, the group author Back@Home Investigators was omitted from the authorship list. Furthermore, the equal contribution note was erroneously assigned to all authors when it should have only been assigned to the first two authors, Alla Melman and Min Jiat Teng.

The authorship list was originally published as:

Alla Melman*, Min Jiat Teng*, Danielle M Coombs*, Qiang Li*, Laurent Billot*, Thomas Lung*, Eileen Rogan*, Mona Marabani*, Owen Hutchings*, Chris G Maher*, Gustavo C Machado*

*all authors contributed equally

This has been changed to:

Alla Melman*, Min Jiat Teng*, Danielle M Coombs, Qiang Li, Laurent Billot, Thomas Lung, Eileen Rogan, Mona Marabani, Owen Hutchings, Chris G Maher, Gustavo C Machado, Back@Home Investigators

*these authors contributed equally

Then, the *Acknowledgments* section was originally published as follows:

This study has received a Hospitals Contribution Fund (HCF) Research Grant: Optimizing outcomes for patients with back pain by preventing hospital admission (Aus $315,000 [US $207,500]), HCF Health Services Research Grant, HCF Research Foundation. We would like to thank the Back@Home collaborators (Bethan Richards, Ananthila Anandacoomarasamy, Kirsten McCaffery, Miranda Shaw, Cassandra Dearing, Ian Harris, Rachelle Buchbinder, Chris Needs, James Edwards, Narcyz Ghinea, Michael Dinh, Dane Chalkley, and Rachael Dodd) for their assistance with the project planning and governance.

This has been changed to

This study has received a Hospitals Contribution Fund (HCF) Research Grant: Optimizing outcomes for patients with back pain by preventing hospital admission (Aus $315,000 [US $207,500]), HCF Health Services Research Grant, HCF Research Foundation.

The Back@Home Investigators are: Alla Melman, Danielle M Coombs, Qiang Li, Laurent Billot, Bethan Richards, Eileen Rogan, Ananthila Anandacoomarasamy, Owen Hutchings, Min Jiat Teng, Kirsten McCaffery, Mona Marabani, Miranda Shaw, Cassandra Dearing, Ian Harris, Rachelle Buchbinder, Chris Needs, James Edwards, Narcyz Ghinea, Michael Dinh, Dane Chalkley, Rachael Dodd, Chris G Maher, Gustavo C Machado.

The correction will appear in the online version of the paper on the JMIR Publications website on May 16, 2024, together with the publication of this correction notice. Because this was made after submission to PubMed, PubMed Central, and other full-text repositories, the corrected article has also been resubmitted to those repositories.

